# 2707. Complications of Babesiosis in Immunocompromised and Asplenic/Hyposplenic Patients

**DOI:** 10.1093/ofid/ofad500.2318

**Published:** 2023-11-27

**Authors:** Courtney E Harris, Loukas Kakoullis, Muneerah M Aleissa, Esther Arbona Haddad, Andy J Kim, Rebecca Rooks, Bridget Yates, Urwah Kanwal, Natalie E Izaguirre, Jessica S Little, Sarah P Hammond, Mary W Montgomery, Amy C Sherman, James Maguire, Ann E Woolley, Lindsey R Baden, Nicolas C Issa

**Affiliations:** Brigham & Women's Hospital, Boston, Massachusetts; Mount Auburn Hospital, Watertown, Massachusetts; Brigham and Women's Hospital, Boston, Massachusetts; Brigham and Women's Hospital, Boston, Massachusetts; Brigham and Women's Hospital, Boston, Massachusetts; Brigham and Women's Hospital, Boston, Massachusetts; Brigham and Women's Hospital, Boston, Massachusetts; Brigham and Women's Hospital, Boston, Massachusetts; Brigham and Women's Hospital, Boston, Massachusetts; Brigham and Women's Hospital, Boston, Massachusetts; Massachusetts General Hospital, Boston, Massachusetts; Brigham and Women's Hospital / Harvard Medical School, Boston, MA; Brigham and Women's Hospital, Boston, Massachusetts; Brigham and Women’s Hospital and Harvard Medical School, Boston, Massachusetts; Brigham and Women's Hospital, Boston, Massachusetts; Brigham and Women's Hospital, Boston, Massachusetts; Brigham & Women's Hospital, Boston, Massachusetts

## Abstract

**Background:**

Babesiosis is a tick-borne disease frequently encountered in the region of New England in the US, with increasing incidence and complications ranging from hemolysis to end-organ damage and death. Immunocompromised (IC) patients and patients with impaired splenic function are at increased risk of complications and relapse.

**Methods:**

This was a multicenter, retrospective study cohort of adult patients diagnosed with babesiosis from 03/2015 – 03/2023 at two institutions. Patient demographics, diagnostic testing, treatment, and outcomes were obtained from the electronic medical record. We aimed to investigate how patient risk factors, complications, length of therapy, and frequency of relapse inform clinical decisions in IC patients.

**Results:**

We identified 57 IC or asplenic/hyposplenic patients with babesiosis. Median age was 66 (IQR 60–76) and 40 (70%) were male. 45 patients (79%) were IC, 21 (37%) were asplenic, and 9 (16%) were both. The initial diagnostic test was a peripheral blood smear in 38 (67%). 25 (35%) were also co-infected with *Borrelia spp.* 54 patients (95%) received initial treatment with combinations of azithromycin (53, 98%), atovaquone (52, 96%), clindamycin (12, 22%), and quinine (2, 7%) with a median duration of 24.5 days [IQR 11-46]). Median treatment duration was shorter in those who were asplenic only (12 days, IQR 9-25.8) compared to those IC with splenic function (27.5 days, IQR 14-46.8) and those who were IC and asplenic (48 days, IQR 17.8-135.8). 3 patients did not receive treatment against *Babesia*. Prior to therapy discontinuation, 37 (65%) of patients had a documented negative smear, and 12 (21%) were PCR negative. 3 cases (5%) experienced a relapse, with 1 patient remaining PCR positive before treatment discontinuation. 4 patients (7%) died within 90 days of babesia diagnosis, with 1 death (2%) secondary to babesiosis [**Table 1**].

**Figure:**
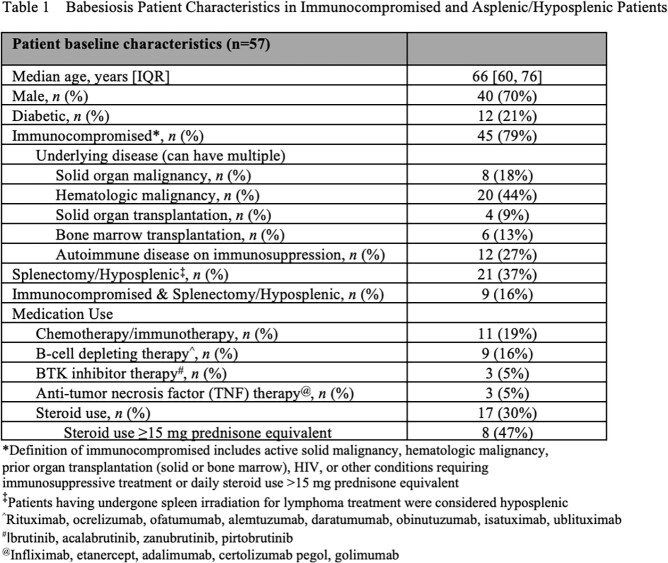

**Figure:**
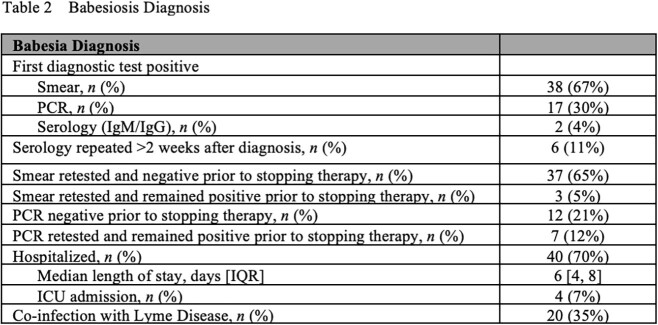

**Figure:**
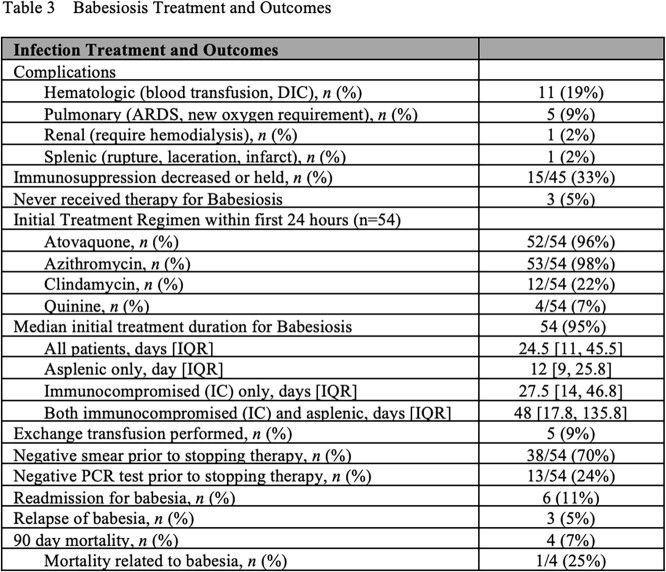

**Conclusion:**

Our analysis demonstrates a low relapse and babesiosis-related mortality rate in this cohort, where IC patients typically receive a prolonged treatment course. Parasitemia detection by blood smear continues to be the initial test to determine disease severity and clearance of parasitemia. Further studies are warranted to determine the optimal length of therapy and criteria for therapy cessation, including the role of PCR.

**Disclosures:**

**Courtney E. Harris, MD**, Dynamed: Advisor/Consultant **Sarah P. Hammond, MD**, F2G: Advisor/Consultant|F2G: Grant/Research Support|GSK: Grant/Research Support|Pfizer: Advisor/Consultant|Scynexis: Grant/Research Support|Seres therapeutics: Advisor/Consultant **Nicolas C. Issa, MD**, AiCuris: Grant/Research Support|Astellas: Grant/Research Support|Boehringer Ingelheim: Advisor/Consultant|Fujifilm: Grant/Research Support|GSK: Grant/Research Support|Merck: Grant/Research Support|Moderna: Grant/Research Support

